# Greenness, Whiteness, and Blueness Assessment With a Novel and Fully Validated HPLC Method for Simultaneous Analysis of Lumacaftor and Ivacaftor in Sweat

**DOI:** 10.1155/ianc/6649147

**Published:** 2026-01-21

**Authors:** Serkan Levent, Abeer Elriş, Saniye Özcan, Nafiz Öncü Can

**Affiliations:** ^1^ Department of Analytical Chemistry, Faculty of Pharmacy, Anadolu University, Eskisehir, 26470, Turkey, anadolu.edu.tr; ^2^ Central Research Laboratory (MERLAB), Faculty of Pharmacy, Anadolu University, Eskisehir, 26470, Turkey, anadolu.edu.tr; ^3^ Department of Analytical Chemistry, Graduate School, Anadolu University, Eskisehir, 26470, Turkey, anadolu.edu.tr

**Keywords:** blueness, HPLC, ivacaftor, lumacaftor, sweat

## Abstract

Sweat is an alternative biological fluid to plasma, urine, hair, and saliva, and it is promising for various pharmaceutical research types. Excessive sweating is one of the symptoms of cystic fibrosis, a hereditary disease. In this study, an easy, simple, applicable, and economical HPLC method was proposed for sweat analysis of the lumacaftor/ivacaftor combination used in the treatment of the disease. The solvent for the method was selected using the Green Solvent Selection Tool (GSST). The mobile phase was gradient elution mode and contained a mixture of 0.1% formic acid in acetonitrile (*v/v*) and 0.1% formic acid in water (*v/v*). Analytes were detected at a wavelength of 220 nm. LOD values for LUMA and IVA are 3.16 and 0.92 μg/mL, respectively. The linearity range was 60–150 μg/mL for both analytes, and matrix‐matched calibration was performed. The greenness was evaluated with AGREE and ComplexGAPI, the whiteness with the red–green–blue 12 (RGB 12) algorithm, and the blueness with the Blue Applicability Degree Index (BAGI). The AGREE score of the method was calculated as 0.72, the BAGI score as 87.5, and the RGB 12 algorithm as 88.3. As a result, the method was presented to researchers as a sustainable, green, and efficient method.

## 1. Introduction

**Figure 1:**
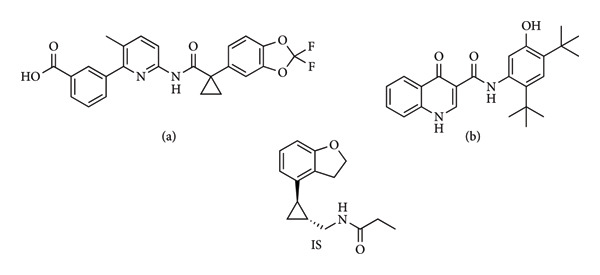
The chemical structures of LUMA (a), IVA (b), and Tasimelteon (IS).

Lumacaftor (LUMA) and ivacaftor (IVA) (Figure 1), also known as cystic fibrosis transmembrane conductance regulator (CFTR) modulators, have been researched for the treatment of patients with certain mutations. LUMA’s effect of folding proteins right and attaching them to the surface of the cell for a long time, and IVA’s effect of keeping Cl canals open, occur as synergistic effects [1]. These are found to be effective in mitigating the symptoms of people who have a mutation of F508del [2]. The use of these two agents together significantly reduces the exacerbation of the disease and reduces the need for hospitalization using intravenous antibiotics [3]. A study using the combination of LUMA and IVA found that the lung clearance index rate was significantly reduced and another study demonstrated an increase in the FEV1 value (the value indicates how healthy the lungs are working) [4].

The USA Food and Drug Administration approved LUMA and IVA for use by patients with the F508del mutation in 2015 and 2012, respectively. A lot of studies are made in order to guide pharmacokinetics, clinical, cytotoxic, and toxicological studies on the cell medium of human plasma [5]. In addition, research on sputum and sweat is very popular because those samples are noninvasive, so taking a sample is easy. (There is less need for purification, precipitation, and precipitation stages because they contain fewer protein ingredients compared with the other samples.) There have been high‐quality new analytical studies in recent years, whereas the literature knowledge is still limited for further research.

For simultaneous LUMA and IVA analysis in biofluids, plasma, and sputum, HPLC [5, 6] and LC–MS/MS analytical techniques are available for the analysis of cord blood and breast milk [5, 7]. Additionally, analysis methods have been suggested or used separately or with different compounds in various clinical and pharmacokinetic studies [8–11]. Among these, blood analyses are invasive, a source of stress, and tiring for the patient during long‐term follow‐up. Collecting urine and saliva samples is a common, noninvasive method. However, as a biofluid, it comes to the fore with potential safety problems. Sweat, another noninvasive sample type, is seen as one of the most important bioanalysis sources of the future [12]. Especially in diseases where abnormal sweating occurs, such as in cystic fibrosis (CF) patients, samples can be easily taken without tiring the volunteers or exposing them to any stress. This may perhaps pave the way for different bioanalytical applications through wearable devices in the future.

Sweat is produced by three types of sweat glands: eccrine, apocrine, and apoeccrine glands. Eccrine glands are glands distributed over the entire surface that open freely to the epidermal surface. Apocrine and apoeccrine are found in hairy areas of the body and open into hair canals. Today, sweat secreted from eccrine sweat glands is used for analysis. Apocrine sweat was also used in this study. Eccrine sweat contains 99% water and 1% hormones, electrolytes, metabolites, peptides, and proteins. Apocrine sweat is similarly water‐based although it is oil‐based with protein, lipid, steroid, and sebum content [12, 13].

Sweat is one of the most important sample sources for various drugs, prohibited substances, and clinical evaluations. It has many advantages for the analyst, such as the influence of stress and external factors, situational changes, and easy sampling. It is also easier and less tiring for both the patient and the volunteer. CF is a hereditary disease, and there are many factors that need to be considered and studied. LUMA/IVA is one of the most promising drugs because it reduces hospital stays and allows use in children. In this study, a method for the analysis of LUMA and IVA in sweat by HPLC was developed that is easy to apply, fast, sensitive, selective, and has high accuracy and precision. In addition, the developed method was evaluated in detail according to the National Environmental Methods Index (NEMI), analytical eco‐scale, Analytical GREEnness Metric (AGREE), and Green Analytical Procedure Index (GAPI).

## 2. Material and Methods

### 2.1. Reagents and Chemicals

The standard substances of LUMA and IVA were purchased from TRC Company (Canada) with a purity of 99.9% (*w/w*). Acetonitrile and formic acid were procured from Sigma‐Aldrich Chemie GmbH (Germany). Tasimelteon from also Sigma‐Aldrich Chemie GmbH (Germany) was selected as the internal standard due to its similar spectroscopic response and its reasonable similarity to the analytes in terms of physicochemical properties.

### 2.2. Instrumentation

The chromatography procedure was conducted utilizing a Prominence series of HPLC manufactured by Shimadzu Co. in Japan. The instrument comprised an LC‐20AT tandem dual‐plunger pump that was equipped with a low‐pressure gradient unit, a CTO‐10ASVP column oven, a SIL‐20AC HT auto‐sampler, a CBM‐20A communications bus module, a DGU‐20A5R online degasser, and an SPD‐M20A photodiode array detector (PDA). The instrument’s dwell volume was approximately 500 μL.

The additional laboratory instruments utilized in the study included the RK 100 H model ultrasonic bath manufactured by Bandelin (Germany), the XSE 105 Dual Range model analytical balance and the SevenMulti model pH meter produced by Mettler‐Toledo (Switzerland), the Rotina 380R model cooling centrifuge manufactured by Hettich (Germany), and the Heidolph Reax Top model vortex mixer (Germany).

### 2.3. Chromatographic Parameters

Throughout the study, a mobile phase gradient elution was employed. The mobile phase contained a mixture of 0.1% formic acid in acetonitrile and 0.1% formic acid in water. The gradient elution program is provided in Table 1. Chromatographic analyses were performed on an HPLC column 10 cm × 4.6 mm, 2.7 μm, Ascentis® Express Phenyl‐Hexyl (USA). The injection volume was 2 μL, and the column oven temperature was set at 40°C. The flow rate was 1.0 mL/min, and the PDA detector was set at a wavelength of 220 nm.

**Table 1:** The gradient elution program of the proposed method.

Time (min)	Mobile phase (%ACN)
0.0	40
0.0–1.0	40
1.0–2.0	70
2.0–2.5	50
2.5–3.0	40
3.0–4.0	30
4.0–4.5	40
4.5–5.0	50
5.0–6.0	60
6.0–6.5	40
8.00	—

### 2.4. Preparation of the Stock Solutions

Stock solutions were prepared by dissolving LUMA and IVA as analytes, with the IS, separately in acetonitrile. To prepare the analytes and the IS solution, 1.00 mg of each substance was weighed and then dissolved in 1.00 mL of acetonitrile.

For the mixture of LUMA, IVA, and the IS, 50 μL of the standard solution for each substance was transferred to a vial using a micropipette and thoroughly mixed. Additional dilutions for calibration solutions were prepared by making necessary dilutions for these stock solutions using ACN.

### 2.5. Preparation of the Sample Solutions

Sweat samples were collected from a volunteer with excessive sweating (hyperhidrosis). The individual was collected between 13:00 and 16:00 at normal room temperature. The samples were collected on 3 different days to be independent of various biological activities.

The sweat sample was prepared as follows: 100 μL of the sweat sample was taken, and then, it was diluted to 1 mL with 900 μL of acetonitrile and filtered through a nonsterile membrane filter (47 mm i.d., 0.22 μm pore size, Sartorius, Germany). Concentrations of 60.0, 75.0, and 90 μg/mL were prepared by adding stock solutions of LUMA, IVA, and IS to the sweat sample.

### 2.6. Preparation of Mobile Phase Solutions

Chromatographic separation was carried out using a gradient elution mode, which involves altering the composition of the mobile phase. The mobile phase mixture was prepared by combining two solutions: 0.1% formic acid in acetonitrile and 0.1% formic acid in water. Both polar and nonpolar mobile phases were prepared by adding 1 mL of formic acid to 1 L of the respective solvent. The resulting solutions were then sonicated for 15 min.

### 2.7. Validation of the Method

In the developed method, method validity was established in accordance with the ICH guidelines Q2 (R1), demonstrating that the method met the required analytical criteria. Linearity, precision, selectivity, accuracy, detection limit, quantification limit, and system suitability tests (SSTs) were all conducted to assess and validate the method [14].

SSTs are established during the analysis to ensure the transferability of the current system to a method‐validated system, to maintain the smooth operation of the system, and to avoid unnecessary chromatographic efficiency. These criteria typically include parameters such as the theoretical number of plates (*N*), resolution (*R*_*s*_), tailing factor (*T*), selectivity factor (*α*), and capacity factor (*k*′). Each of these parameters was calculated according to the United States Pharmacopeia (USP) method [15].

#### 2.7.1. Stability

Stability studies were carried out that a stock solution of LUMA, IVA, and IS mixture was appropriately diluted in acetonitrile (75 μg/mL) and analyzed periodically over a span of three days at −20°C, subjected to three freeze–thaw cycles, to evaluate the stability of both the solution and the mobile phase. Another stability test was performed by examining the stability of the mixture in laboratory conditions for 2 days.

#### 2.7.2. Linearity

To assess the linearity of the developed HPLC method, standard solutions of LUMA and IVA were prepared and analyzed in acetonitrile at six different concentration levels ranging from 25% to 200% (18.75, 60, 75, 90, 112.5, and 150 μg/mL). The analyses were conducted with a minimum of three repetitions in the detector system, and calculations were performed using the average of the obtained areas. Linearity was examined through three repetitions, both intraday and interday, and the resulting linear regression analyses were evaluated.

#### 2.7.3. Accuracy

To assess accuracy, recovery studies were conducted. The prepared standard solutions of LUMA and IVA were used at three different concentration levels: low (60 μg/mL), medium (75 μg/mL), and high (90 μg/mL). Three independent sets were prepared for each concentration level, and parameters such as the standard deviation (SD), relative standard deviation (%RSD), and mean recovery with a 95% confidence interval were calculated for the results.

#### 2.7.4. Precision

The solutions at a concentration of 75.0 μg/mL (100% of the standard solutions used in the method) were prepared and analyzed over three consecutive days with *n* = 3 replicates. Intermediate precision studies were carried out, and the results were statistically evaluated using analysis of variance. The analysis included calculating the mean, SD, mean standard error, and %RSD at 95% confidence level.

#### 2.7.5. Limit of Detection (LOD) and Limit of Quantification (LOQ)

The LOQ and LOD values were determined visually following the guidelines outlined in the ICH guide [14].

#### 2.7.6. Robustness

The robustness of the method was evaluated using standard solutions of LUMA and IVA, each corresponding to a concentration level of 100%. Deviations in various system suitability parameters were calculated by varying the flow rate, the percentage of the nonpolar component of the mobile phase, the column temperature, and the amount of the organic component of the mobile phase by ±10%.

## 3. Results and Discussion

### 3.1. Method Development

The hydrophobic structure of the CFTR modulators LUMA and IVA led to the preference for reverse‐phase chromatography in the development of the HPLC method. The log*p* values of LUMA and IVA are between 5 and 5.76 [16]. A small particle (2.7 μm) column was chosen to provide high resolution, increase analytical sensitivity, and elute in a shorter time. We tested columns with the same particle size and properties, but with different functional groups (F5, C18, and Phenyl‐Hexyl). The F5 column was tested first and showed poor separation of the LUMA peak. Upon testing the C18 column, the elution time was too long. It was concluded that the insufficient and weak interactions between the F5 column and the analytes were responsible for this outcome. In the case of the C18 column, the nonpolar interior of the column and the high nonpolarity of the analytes led to strong interactions. The interactions were so intense that even acetonitrile, the organic component of the mobile phase, could not provide an effective and appropriate elution. Finally, it was observed that effective and efficient separation could be achieved when the column with the Phenyl‐Hexyl functional group was used. Of course, for this purpose, gradient was chosen as the appropriate elution mode by optimizing the mobile phase composition. When the gradient elution mode was optimized, truly excellent separation, minimal tailing factor, and a short elution time were achieved.

The method can elute LUMA and IVA in a short time (8 min), as shown in Figure 2. The log*p* values of LUMA and IVA molecules show that they have similar hydrophobicity profiles. This similarity suggests that their retention and partitioning between two phases of the HPLC system are both possible and likely to be similar. The optimal chromatographic resolution was achieved by utilizing a mobile phase comprising acetonitrile containing 0.1% formic acid and water containing 0.1% formic acid and employing a gradient elution (as outlined in Section 2). The retention times of LUMA, IVA, and IS were 6.69, 4.20, and 2.51 min, respectively. As given in Figure 2, there was no interference with the retention times of either analyte in blank samples, nor was there any interference detected when LUMA and IVA were simultaneously analyzed.

Figure 2Assay chromatograms of IVA (LOQ), LUMA (LOQ), and IS (75 μg/mL) (a), and blank samples (b).

**Figure:**
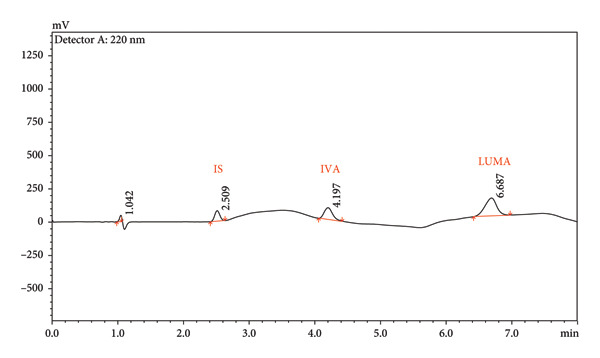
(a)

**Figure:**
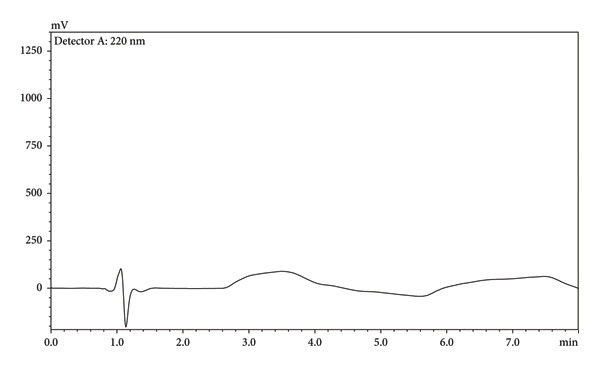
(b)

The results of all SST, as specified by the ICH Q2 (R1) guideline for the optimum method condition, were computed after a thorough analysis [14]. The results are presented in Table 2. All SST values are within the limits and respect the rules for chromatographic separation.

**Table 2:** The system suitability data for LUMA (75 μg/mL), IVA (75 μg/mL), and IS (75 μg/mL).

Parameter	LUMA	IVA	IS
Retention time (min)	6.687	4.197	2.509
Relative retention time (min)	2.49	1.69	—
Relative standard deviation (%) of retention time	0.09	0.16	—
Precision for area	25731.9	14885.2	7643.9
Precision for relative area	0.07	0.03	—
Injection precision for retention time (min)	0.002	0.003	0.001
Capacity factor (*k′*)	5.39	3.01	1.41
Theoretical number of plates (*N*)	18460.8	15055.3	12020.9
Resolution (*R*_*s*_)	8.74	8.57	12.84
Tailing factor(*T*)	0.94	1.38	1.14
USP width^∗^	0.34	0.23	0.16
HETP	24.03	28.96	36.13

^∗^United States Pharmacopeia.

### 3.2. Method Validation

Sweat is a relatively easy matrix, although it depends on the method type and analyte to be analyzed. As a diagnostic and detection fluid, it has many advantages over other biological fluids [17]. Monitoring the pharmacokinetic profiles of drugs in sweat has made significant progress, particularly in biomedical applications [18]. However, it is useful to pay attention to the relationship between blood concentration and sweat concentration [19]. In addition, LOD, LOQ, and working range values in blood plasma should be lower. However, this approach offers advantages such as reduced protection against biofouling and improved selectivity. However, it is evident that further study and research are necessary for the analysis of biologically based analytes.

While validating the method, no significant difference was observed between the matrix‐paired calibration method and the standard calibration method. In general, the validation procedure was successful and efficient. Naturally, the physical and chemical properties of the analytes and the selected IS significantly influence this process. All of them exhibit stability and spectroscopic properties that align with the method under development. Additionally, no interference, steric effects, or stability problems from the matrix environment were observed. Data and observations of all validation parameters examined are given in detail in the following sections.

To conduct linearity and accuracy tests, the ratio between the peak area and retention time of each standard solution and the IS solution contained in that solution was determined. A linear calibration range was established, ranging from 60 to 150 μg/mL. The precision study was evaluated at the intraday and interday levels for 3 days. The results are shown in Table 3.

**Table 3:** Statistical data for the linearity and precision of LUMA and IVA.

	Parameter	LUMA	IVA
Intraday(*k* = 3)	Linearity range	60–150 (μg/mL)	60–150 (μg/mL)
	Slope (intraday, *n* = 9)	2.0406	0.8642
	Intercept (intraday, *n* = 9)	−0.2806	−0.1246
	Regression coefficient (intraday, *n* = 9)	0.9981	0.9995
	SD of slope	0.0518	0.0260
	SD of intercept	0.1582	0.0355
	ANOVA	F(2.24) = 0.0002 *p* = 0.996 (*p* > 0.05)	F(2.24) = 0.0004 *p* = 0.998 (*p* > 0.05)

	LOD (μg/mL)	0.92	3.16

	LOQ (μg/mL)	2.81	5.08

Interday (*k* = 3)	Slope (interday, *n* = 27)	2.0447	1.3023
	Intercept (interday, *n* = 27)	−0.3301	−0.2073
	Regression coefficient (interday, *n* = 27)	0.9952	0.9970
	SD of slope	0.1828	0.0929
	SD of intercept	0.2497	0.1269
	ANOVA	F(2.24) = 0.005 *p* = 0.761 (*p* > 0.05)	F(2.24) = 0.009 *p* = 0.656 (*p* > 0.05)

Stability studies were conducted by mixing LUMA and IVA into the working solutions and adding the IS. Short‐term stability and three freeze–thaw cycles were included in the stability studies. According to the results obtained, it can be seen in Table 4 that LUMA and IVA are quite stable under operating conditions.

**Table 4:** Stability tests for LUMA and IVA.

	Added concentration	Short‐term stability (24 h, room temperature)		Short‐term stability (48 h, room temperature)		Freeze–thaw stability (3 cycles)	
		Found (mean)	SD (95% CI^∗^)	Found (mean)	SD (95% CI^∗^)	Found (mean)	SD (95% CI^∗^)
LUMA	75 μg/mL	74.89	1.04	74.24	0.08	74.25	3.30
IVA	75 μg/mL	77.18	2.22	77.16	1.67	78.92	0.94

^∗^at the 95% confidence interval.

Moreover, the robustness studies were conducted by altering the effective factor, which may vary from one instrument to another, and observing the variations in the system suitability parameters values and calculating the SD. As part of robustness analyses, modifications were made to flow rate, column temperature, organic phase ratio, and formic acid percentage. The effects of these changes on retention time, recovery, capacity factor, resolution, theoretical plate number, and tailing factor were examined. The obtained results are presented in Table 5.

**Table 5:** The results of robustness studies for LUMA (75 μg/mL) and IVA (75 μg/mL).

Parameter		% Difference of RT (mean ± SD)	% Recovery (mean ± SD)^∗^	% Difference of capacity factor (mean ± SD)	% Difference of resolution (mean ± SD)^∗^	% Difference of number of theoretical plates (mean ± SD)^∗^	% Difference of tailing factor (mean ± SD)^∗^
*LUMA*							
Flow rate (mL/min)	0.90	−1.05 ± 0.25	−17.81 ± 3.53	−0.49 ± 0.57	−30.27 ± 6.85	6.57 ± 5.89	4.46 ± 2.81
	1.10	7.92 ± 0.25	−88.06 ± 0.85	9.49 ± 0.34	34.15 ± 12.14	26.03 ± 171.16	−8.40 ± 14.74
Column temperature (°C)	36	0.77 ± 0.31	−10.49 ± 3.56	2.29 ± 0.32	2.79 ± 1.75	−47.39 ± 1.29	13.91 ± 2.74
	44	−0.50 ± 0.13	−0.67±‐31.30	−0.20 ± 1.96	5.12 ± 3.46	−64.82 ± 4.88	10.15 ± 4.37
Percentage of organic phase (%)	27.0	53.42 ± 1,6	−18.06 ± 8.55	96.11 ± 2.36	−40.90 ± 2.18	−93.41 ± 0.66	1.30 ± 28.37
	29.0	−31.43 ± 0.12	−4.87 ± 4.16	−50.37 ± 0.15	−86.70 ± 0.08	6.09 ± 6.50	50.59 ± 8.29
Percentage of formic acid (%)	0.09	−3.04 ± 0.37	59.58 ± 9.88	1.24 ± 0.57	17.70 ± 2.60	−37.65 ± 2.72	−3.94 ± 0.73
	0.11	−0.34 ± 0.08	36.27 ± 4.80	1.47 ± 0.46	24.80 ± 2.29	−7.10 ± 3.02	5.90 ± 1.63

*IVA*							
Flow rate (mL/min)	0.90	6.46 ± 2.26	33.37 ± 1.88	10.07 ± 2.75	−25.61 ± 8.25	−66.11 ± 43.08	−20.47 ± 5.32
	1.10	1.27 ± 0.13	218.07 ± 16.28	11.38 ± 16.38	31.74 ± 1.23	163.74 ± 341.33	−13.46 ± 2.05
Column temperature (°C)	36	0.66 ± 0.44	−5.079 ± 1.60	2.47 ± 0.42	−10.40 ± 2.77	−76.81 ± 1.73	1.67 ± 9.77
	44	0.51 ± 0.40	−5.37 ± 1.03	−0.23 ± 0.37	15.63 ± 1.73	−48.37 ± 2.50	−10.80 ± 3.30
Percentage of organic phase (%)	27.0	32.85 ± 1.02	−29.84 ± 21.65	75.72 ± 1.65	7.51 ± 7.06	−85.65 ± 2.20	−80.96 ± 37.18
	29.0	5.79 ± 0.14	189.17 ± 1.60	−15.03 ± 0.19	42.17 ± 1.09	30.34 ± 4.64	−14.77 ± 2.58
Percentage of formic acid (%)	0.09	−2.82 ± 0.34	338.28 ± 15.73	2.31 ± 0.18	13.69 ± 2.19	−55.36 ± 2.20	−17.61 ± 1.75
	0.11	1.40 ± 0.29	360.88 ± 20.39	3.34 ± 0.43	−0.20 ± 2.53	−73.52 ± 2.22	−21.20 ± 2.50

^∗^%95 confidence interval.

It was used in the conventional addition procedure to conduct recovery tests. We used a sample of sweat to accomplish this goal. The working solution was introduced after the sample was diluted with acetonitrile. Thereafter, the real sample solutions were made at three different concentration levels. For each concentration, RSD values were lower than 5%. The results are shown in Table 6.

**Table 6:** Statistical evaluation of recovery studies performed.

	Added concentration (μg/mL)	Measured concentration (μg/mL)	Recovery (%)	SD	RSD (%)	Error (%)	Mean recovery (%)
IVA	60	63.48	105.81	1.55	2.44	+5.81	102.76
	75	75.35	100.47	2.37	3.15	+0.47	
	90	91.79	101.99	2.37	3.15	+1.99	

LUMA	60	61.53	102.54	2.85	4.63	+2.54	100.99
	75	75.41	100.55	3.70	4.91	+0.55	
	90	89.91	99.90	0.87	0.97	−0.10	

Various clinical studies have shown that sweat can be used as an alternative biological fluid in therapeutic drug monitoring. It is also thought that molecular properties, hydrophobicity, and physiological conditions affect sweat/plasma drug correlation as important factors [18]. However, it is an important requirement to specifically examine the relationship between sweat and plasma concentration according to drug type and disease [20, 21]. Although there are studies in the literature on therapeutic monitoring of different drug types in sweat, there are no studies on patients with CF or the LUMA/IVA combination. As mentioned in the introduction, a method has been proposed for HPLC analysis in plasma and sputum [5]. However, since the LC–MS/MS method was also recommended in the study, the authors focused more on it. For HPLC, LOD and LOQ information is given as working range 1 to 80 μg/mL. Even though the linear range in this article is at a higher concentration, the LOQ and LOD concentrations were lower. Additionally, the method was fully validated. Finally, it is noteworthy that sensors are promising and there will be more work to be done on this subject. However, the importance of chromatographic methods cannot be denied for routine analyses to obtain data with high accuracy and precision.

### 3.3. Method Greenness, Whiteness, and Blueness

The sustainable, economical, efficient, and environmentally friendly features of the developed method were examined in detail and comprehensively. For this purpose, tools called the Green Solvent Selection Tool (GSST), the AGREE as a quantitative tool for analytical green chemistry (GAC) of the method, and the Complementary Green Analytical Procedure Index (ComplexGAPI) as a semiqualitative tool were used to select the appropriate solvents. The red‐green‐blue (RGB) 12 algorithm was preferred for the whiteness evaluation of the method. Finally, the Blue Applicability Grade Index (BAGI) tool was used to determine the blueness of the method. The obtained data and observations are presented in detail in the following section.

#### 3.3.1. GSST

**Figure 3:**
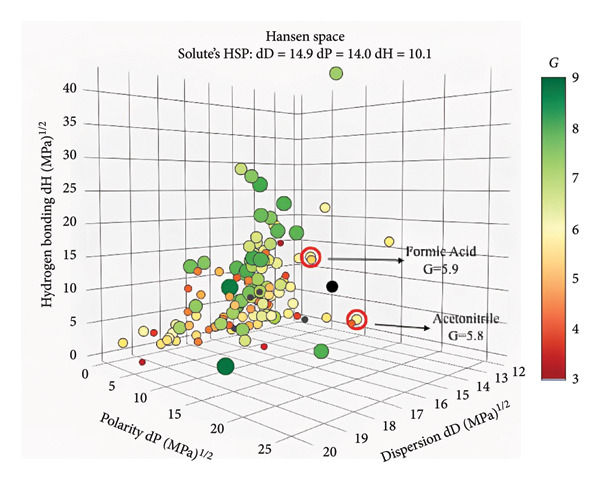
(*G*) Scores by GSST for solvents of the proposed method.

GSST, which provides a broad and comprehensive evaluation in solvent selection, is a highly preferred tool [22]. It evaluates the solvent not only as green but also as “sustainable,” in many aspects such as analytics, availability, impact on human health, and the environment. Therefore, it is a useful and comprehensive tool. GSST is a 3D chart that includes the evaluation of many solvents. In the graph, if the solvent has a large green circle (*G* ≥ 7), it means that it is highly sustainable, and a medium‐sized yellow/orange circle means that it is a solvent with limited sustainability (*G* = 5–6). The solvent with a small red circle is one that should not be preferred and is *G* ≤ 4. In this study, the scores obtained from the GSST tool for the solvents preferred according to both method conditions and sustainability are given in Figure 3. Formic acid and acetonitrile were preferred in the mobile phase component and sample preparation as solvents. The *G* scores of formic acid and acetonitrile are 5.9 and 5.8, respectively. The solvents of the method have limited sustainability.

#### 3.3.2. The Greenness of the Proposed Method

Environmental awareness initially led to the introduction of GAC techniques. However, over time, the consideration of analytical approaches and the limitations arising from the nature of the methods has transformed this trend into a focus on the sustainability of analytical methods. GAC is an evaluation of how much of the created method’s 12 principles overlap [23]. Here, the method’s limitations and needs are as guiding as the researcher’s preferences. The evaluated limitations and approaches to GAC principles in this study were AGREE and ComplexGAPI tools. AGREE is a tool that is simple, flexible, and provides mathematical results that adhere to all 12 principles of GAC [24, 25]. It also has software that asks the researcher about each principle’s weight. ComplexGAPI is a five‐pentagram pictogram with an additional hexagon [26] and provides semiquantitative data. It assesses the overall duration of the analytical methodology, encompassing the initial stages, the process, and the outcome. Figures 4(a) and 4(b) present the AGREE and ComplexGAPI pictograms of the method, respectively. The weak points on the AGREE scale are energy use and renewable solvent, numbers 7 and 12, respectively. According to the scale, a score of 0.60 or higher is considered green. Therefore, a score of 0.72 can be considered perfect greenness. On the ComplexGAPI scale, the method’s weak points are the National Fire Protection Association (NFPA) scores of the solvents used and the amount of waste obtained in the HPLC system. The recommendation against a recycling method for the resulting waste represents another weak point. In addition to this, the pictogram also includes the NFPA scores of the solvents used in the preanalysis step, represented by yellow regions. As a result, it appears that the method is green, cost‐effective, and highly efficient.

Figure 4AGREE (a), ComplexGAPI (b), BAGI (c), and RGB 12 algorithm (d) metrics of the proposed method.

**Figure:**
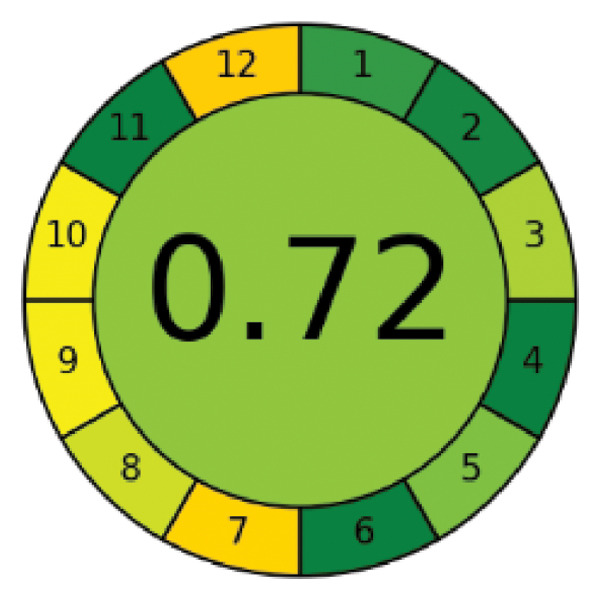
(a)

**Figure:**
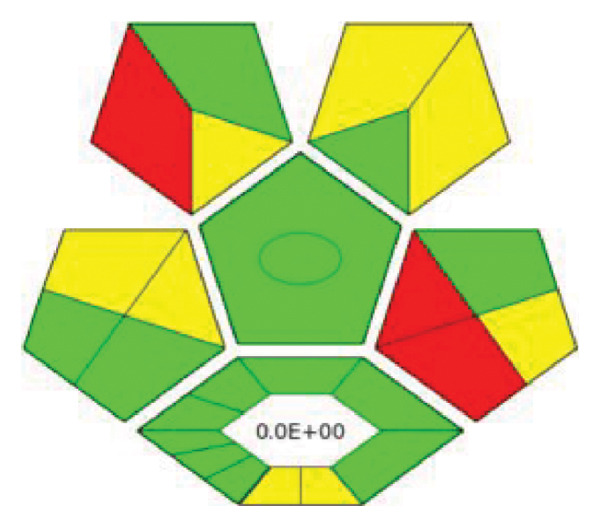
(b)

**Figure:**
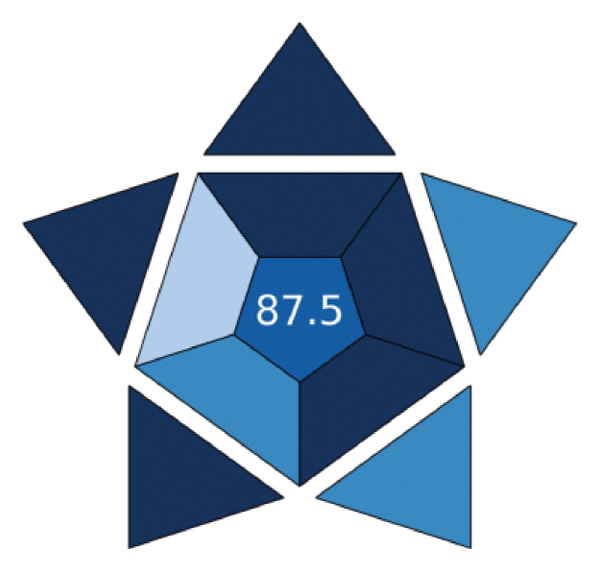
(c)

**Figure:**
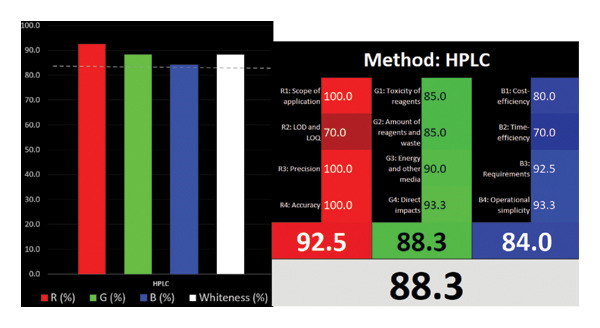
(d)

#### 3.3.3. The Blueness of the Proposed Method

The BAGI of the method is evaluated by considering the analytical difficulties. It evaluates the method with only 10 analytical parameters. Each of these 10 criteria pertains to the method’s efficiency within the current analytical capacity. Every laboratory can access the method’s device, chemical, and preparation stages, which reveal complete performance features such as the analyte and the number of samples per unit time [27]. The method serves as both a quantitative and qualitative tool, enabling the BAGI metric to yield two distinct outcomes. The pictogram’s color tone (closeness to dark blue) indicates whether it fits the specified criteria, and its score indicates its performance. The closer it is to 100, the better the performance. The developed method’s BAGI metric appears in Figure 4(c). The method uses HPLC, a readily available device, resulting in a short analysis time and a simple sample preparation method. The pictogram is dark blue, and the calculated performance score is 87.5. This pictogram proves that the method is very practical and applicable, and visually reveals it weaknesses and strengths.

#### 3.3.4. The Whiteness of the Proposed Method

It is a combined tool that uses whiteness to indicate adherence to the GAC guidelines of the analytical chemistry method, red to indicate method performance, and blue to indicate productivity, practicality, and economy. The RGB 12 algorithm has green, red, and blue colors reflecting three principles [28]. The most advantageous aspect of this tool is that it also considers method validation criteria. The approach involves scoring and coloring each evaluation. The higher the saturation of the three colors, the higher the score, and the closer the method is too white. Figure 4(d) presents the RGB 12 algorithm for the presented method. As a result, the technique has almost reached saturation for all three colors. The points where the colors are darker, that is, weaker, are seen to be toxicities of chemicals with LOD and LOQ concentrations. The authors gave this score for the red color because they believed that lower LOD and LOQ values were required due to the sample’s sweatiness. Considering the entire cartel, the method’s whiteness score is 88.3, which is a very good value.

### 3.4. Application

Hereditary diseases have a very different place from other diseases, both pharmacologically and clinically. Patients’ treatment aims to reduce these effects rather than heal them. In the future, CF will affect many more people than it does today. Therefore, every step that will make the lives of people with hereditary diseases easier is very important. Blood tests are a factor that affects patients’ lives and increases the burden during routine checks. New studies have shown that the symptom of excessive sweating in CF patients is quite distinctive and varies depending on factors such as age and gender [29, 30]. Therefore, this disadvantage can be turned into an advantage or a benefit during the treatment process. This is the primary concept and goal of our research. Our aim is to develop an analysis method that can support various pharmacological and pharmacokinetic studies and offer a different option. For this purpose, it is to develop a fast, easy, and simple method that is fully validated and whose efficiency and sustainability are demonstrated in detail. The study’s weakness lies in the inability to analyze the developed method in patients undergoing the actual IVA/LUMA combined treatment. Despite the authors’ great efforts and requests, legal procedures prevented this from being possible. Of course, it is every researcher’s dream to see the applicability of the method they developed on real samples. Despite all these negativities, all the parameters that should be included and examined in the developed method were studied in detail.

## 4. Conclusion

To sum up, in the paper, the first time an easy, simple, fast, selective, green, white, blue, and economical chromatographic approach for the simultaneous determination of LUMA and IVA in sweat is presented. Sweat samples were collected from a volunteer subject with hyperhidrosis in the afternoon under room conditions. The selectivity, precision, and accuracy of the method were studied under these sample conditions. In addition, the method has a simple sample preparation procedure, and it has excellent recovery data. The method is stable, robust, fully validated, and analytically accurate. The method’s weakness is its inapplicability to CF patients undergoing Orkambi® treatment. Although the researchers made the necessary efforts in this regard, they did not able to progress owing to ethical restrictions. Moreover, the sustainability, efficiency, greenness, and cost‐effectiveness of the developed method were evaluated with 5 metric tools: GSST, AGREE, ComplexGAPI, RGB 12, and the current BAGI. The method can be efficiently applied for various pharmacokinetic and drug monitoring studies due to its noninvasive nature. Finally, the pioneer paper is original in terms of content and concept and will offer many researchers different perspectives in terms of pharmaceutical analyses in sweat. In future work, the developed method will be adapted to an LC–MS platform, and the resulting data will be compared with our current findings.

**Nomenclature**
AGREE: Analytical GREEnness Metric
BAGI: Blue Applicability Grade Index
ComplexGAPI: Complementary Green Analytical Procedure Index
GSST: Green Solvent Selection Tool
ICH: International Council on Harmonization
IS: Internal Standard
LUMA: Lumacaftor
IVA: Ivacaftor
RGB 12: Red‐green‐blue 12
SST: System suitability test

## Conflicts of Interest

The authors declare no conflicts of interest.

## Funding

The study was funded by Anadolu Üniversitesi, 2005S039.

## Data Availability

The data that support the findings of this study are available from the corresponding author upon reasonable request.
